# COVID-19 Vaccines: Ensuring Social Justice and Health Equity among Refugees in Africa

**DOI:** 10.5334/aogh.3415

**Published:** 2021-11-03

**Authors:** Emery Manirambona, Oliver Hague, Luiza Farache Trajano, Annabel Killen, Laura Wilkins, Menelas Nkeshimana, Don Eliseo Lucero-Prisno III

**Affiliations:** 1 College of Medicine and Health Sciences, University of Rwanda, Kigali, Rwanda; 2 Medical Sciences Division, University of Oxford, Oxford, United Kingdom; 3 Department of Anesthesia, Emergency Medicine and Critical Care, Centre Hospitalier Universitaire de Kigali and University of Rwanda, Kigali, Rwanda; 4 Department of Global Health and Development, London School of Hygiene and Tropical Medicine, London, United Kingdom

## Abstract

COVID-19 poses a particular threat to refugees in Africa. Overcrowded living conditions and lack of effective sanitation make refugees highly vulnerable to infection. Furthermore, migration has the potential to undermine measures to control viral spread. As a result, vaccination of the refugee community in Africa must be considered key in the vaccination plan to end the worldwide COVID-19 pandemic. Although the WHO has approved vaccines for emergency use worldwide in vulnerable groups through the COVID-19 Vaccines Global Access (COVAX) program, there is a lack of a strategy for achieving vaccination in the African refugee population. A specific strategy for refugee vaccination must be among the top priorities at national, regional, and global levels to ensure all refugees and asylum seekers in African countries have equitable and quality vaccine assistance regardless of displacement, statelessness, and financial hardship. We call on leaders in Africa and worldwide to ensure that refugee vaccination is a priority to protect this highly at-risk population and achieve an end to the current pandemic.

## Viewpoint

COVID-19 has already infected 5,330,048 people in Africa [1], with more than 139,506 deaths as of June 23, 2021 [2]. SARS-COV-2, the virus causing COVID-19, spreads rapidly and thus national and international public health authorities, such as the World Health Organization (WHO), advised the implementation of strict lockdowns, hygiene, and social distancing in order to reduce spread. Although such measures are important to curtail transmission, adherence is particularly difficult in resource-limited conditions. Those reliant on daily income for survival, such as street vendors [3], risk economic devastation as a result of strict lockdowns. Refugees in Africa have been severely affected by the pandemic—both as a direct consequence of infection with poor healthcare access, and indirectly through socioeconomic disruption caused by the pandemic [4].

Key to sustainable solutions to the global COVID-19 pandemic is the development and manufacture of vaccines. COVID-19 Vaccines Global Access (COVAX) was established to ensure equitable worldwide access to COVID-19 vaccines [5]. Since February 2021, an initiative co-led by the Coalition for Epidemic Preparedness Innovations (CEPI), The Global Alliance for Vaccines and Immunizations (GAVI), and the WHO rolled out COVID-19 vaccine doses in some African countries, prioritising frontline health workers and clinically vulnerable groups. However, this initiative has not been as successful as hoped, because of the paucity of vaccines. Despite the threat posed by the highly contagious and lethal Delta variant of COVID-19, which has spread rapidly across the continent since June 2021 [6], some African countries were still awaiting delivery of vaccines to provide a first dose to their vulnerable citizens [6].

Despite the challenges described above, there are examples of some countries prioritising refugee vaccination as part of their public health policy. In March 2021, Rwanda’s government vaccinated 412 refugees. In Rwanda, the first dose of COVID-19 vaccine is being given to high-risk groups such as healthcare workers, elderly people with chronic health conditions, those aged 65+, and other frontline workers. Refugees meeting these criteria are included in the first stage of the vaccination campaign [7].

COVID-19 has exacerbated tremendous health inequalities that exist in Africa, as across the globe, and highlights discrimination faced by refugees. Poor living conditions and extreme poverty make African refugees highly susceptible to SARS-COV-2. Overcrowding and shared facilities make it difficult to comply with social distancing, while poor access to essential sanitation such as water and disinfectant reduce the ability to follow hygiene recommendations [8]. Refugees lacking a permanent residence where they can isolate face added hostility, such as being accused of spreading the virus [9]. Even without the impact of COVID-19, refugees are at higher risk of mental health disorders, at increased prevalence and severity during the pandemic [10]. These factors are further exacerbated by refugees’ reduced access to healthcare when they become ill [11].

Large, marginalized refugee populations contribute to the susceptibility of the whole population to COVID-19. A subnational spatial analysis of COVID-19 transmission in Kenya showed that large populations without access to sanitation and/or living in temporary dwellings, such as refugees, increases the vulnerability of the entire region to COVID-19 [12]. The continued mobility of refugees, more pronounced in North and West Africa [13], also poses a significant challenge to public health authorities collecting pandemic data in their jurisdiction. Ongoing conflicts in certain regions of Africa pose an additional obstacle to public health campaigns attempting to reach refugees [14]. Vaccination of the refugee community in Africa is therefore vital not only to protect an extremely vulnerable group of the population, but also as part of the wider public health response to the COVID-19 pandemic.

Despite the development of effective and safe vaccines and the establishment of COVAX as a body to advance worldwide access to vaccines [5], there exists no strategy for vaccination of the refugee population in Africa. Whilst international bodies such as the WHO, UNCHR, and UNICEF are important in coordinating refugee vaccination, they rely on national partners for the delivery of vaccine programmes. Countries with insufficient or without vaccine doses for their vulnerable citizens struggle to vaccinate refugee populations. Wealthier countries with disproportionally high vaccine access must therefore contribute doses to this effort [15]. Finally, there are significant logistical challenges posed by the delivery of two-dose regimes currently licensed for COVID-19 vaccines (except Johnson & Johnson) to a highly mobile population not registered with national or local healthcare systems [16].

A specific strategy for refugee vaccination must be prioritised at the national, regional, and global level to ensure refugees and asylum seekers in African countries have equitable vaccine assistance, regardless of displacement, statelessness, and financial hardship. The African Union, WHO-Africa, UNHCR, WFP, and other regional or international agencies are well placed to support this population. We call on these multinational leaders and stakeholders to work with national governments in prioritising refugees who fulfil the same criteria as the citizens and other vulnerable groups in Africa in COVID-19 vaccine distribution.

Access to effective healthcare services is a human right, and access for both refugees and other vulnerable groups is a moral imperative, particularly during a pandemic. The WHO reserved 5 percent of vaccines in the COVAX program for emergency use in humanitarian settings [17]. We argue that refugees belong to this category of those who should benefit. Vaccinating refugees protects this vulnerable community and ensures no vulnerable people being left behind.

## Conclusion

This article illustrates the worldwide situation of refugees by taking the example of Africa. Refugees in Africa are at higher risk of COVID-19 infection, and the outbreak in mobile refugee populations poses a significant challenge to the containment of the virus in the continent. Vaccination of African refugees not only saves lives amongst this marginalized group, but also limits the development of SARS-CoV-2 variants that place the whole world at a higher risk. We call upon African leaders and global communities to make the vaccination of refugees in Africa and across the globe a priority.

## Abbreviations

COVID-19: Coronavirus Disease 2019

SARS-CoV-2: Severe Acute Respiratory Syndrome Coronavirus 2

WHO: World Health Organization

COVAX: COVID-19 Vaccines Global Access

CEPI: Coalition for Epidemic Preparedness Innovations

GAVI: The Global Alliance for Vaccines and Immunizations

UNHCR: United Nations High Commissioner for Refugees

WFP: World Food Programme

UN: United Nations

UNICEF: United Nations International Children’s Emergency Fund
